# Supplementary material for: Metacells untangle large and complex single-cell transcriptome networks
Source: BMC Bioinformatics. 2022 Aug 13;23:336. doi: 10.1186/s12859-022-04861-1 (PMC9375201; doi:10.1186/s12859-022-04861-1)
Supplement: Supplementary file 1 — Additional file 1. Fig S1. Metacells are compatible with UMAP visualization. Fig S2. Metacell size and gene coverage distributions. Fig S3. Metacells recover rare cell types. Fig S4. Metacells preserve clustering. Fig S5. Metacells are compatible with unweighted clustering. Fig S6. Clustering of metacells is robust to different ways of building metacells. Fig S7. Metacells are compatible with unweighted differential expression analysis. Fig S8. Differential expression of metacells is robust to different ways of building metacells. Fig S9. Metacells preserve differential expression between conditions. Fig S10. Gating strategy for the flow cytometry analysis of DCs from murine KP1.9 lung adenocarcinoma for one representative sample. Fig S11. Cell type annotation in the Cd8_TILs dataset. Fig S12. Conservation of RNA velocity results in the brain_cells dataset. Fig S13. Conservation of RNA velocity results in the pancreatic_cells dataset. Fig S14. Computational time and memory allocation for RNA velocity. Fig S15. Approximate coarse-graining in SuperCell. Fig S16. Computational time and memory allocation for metacell construction and downstream analyses. Table S1. Datasets used for the analysis. Table S2. Datasets integrated in the TIM_atlas dataset. Table S3. Genes ranked better in differential expression analysis (cDC vs pDC) at metacell level. Table S4. Genes ranked better in differential expression analysis (pDC vs cDC) at metacell level. Table S5. Antibodies used in flow cytometry (Fig. 2f, Additional file 1: Fig. S11). [file 12859_2022_4861_MOESM1_ESM.docx]

**Supplementary Material**

**Metacells untangle large and complex single-cell transcriptome networks**

Mariia Bilous^1,2^, Loc Tran^1,2^, Chiara Cianciaruso^3^, Aurélie Gabriel^1,2^, Hugo Michel^1^, Santiago J. Carmona^1,2^, Mikael J. Pittet^3,4,5^, David Gfeller^1,2,*^

^1^Department of Oncology, Ludwig Institute for Cancer Research, University of Lausanne, Lausanne, Switzerland,

^2^Swiss Institute of Bioinformatics (SIB), Lausanne, Switzerland

^3^Department of Pathology and Immunology, University of Geneva, Geneva, Switzerland

^4^Department of Oncology, Geneva University Hospitals, Geneva, Switzerland

^5^Center for Systems Biology, Massachusetts General Hospital and Harvard Medical School, Boston, MA

***Corresponding author:** [David.Gfeller@unil.ch](mailto:david.gfeller@unil.ch)

**Supplementary Figures**


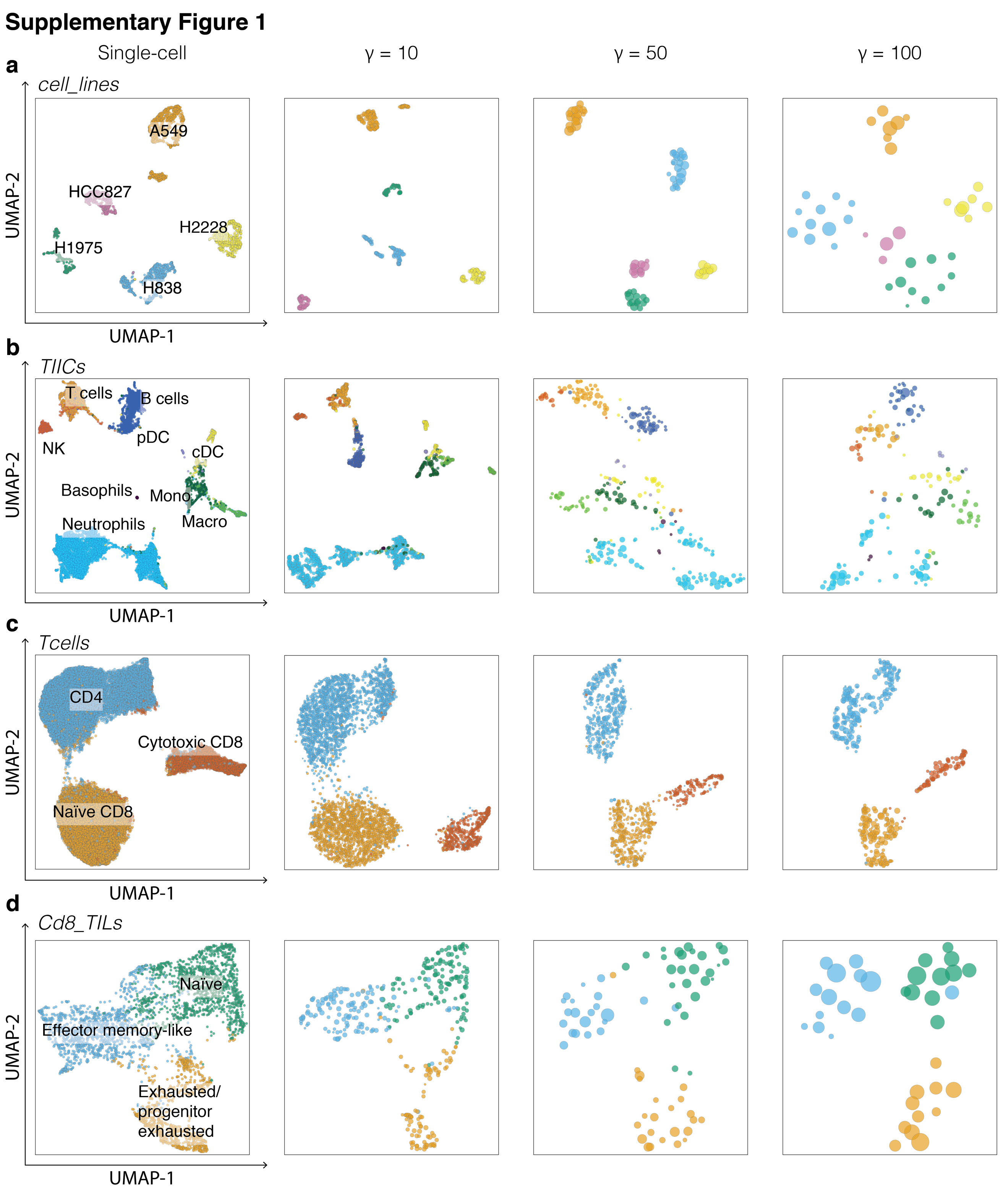


**Supplementary Figure 1. Metacells are compatible with UMAP visualization.**

Examples of UMAPs of metacells at several graining levels. Colors indicate the initial cell type annotation and metacells are colored according to the majority of cells in each metacell. (**a**) Five cancer cell lines (*cell_lines*). (**b**) Tumor-infiltrating immune cells (*TIICs*). (**c**) T cells sorted from PBMC (*Tcells*). (**d**) Tumor-infiltrating CD8 T lymphocytes (*Cd8_TILs*).


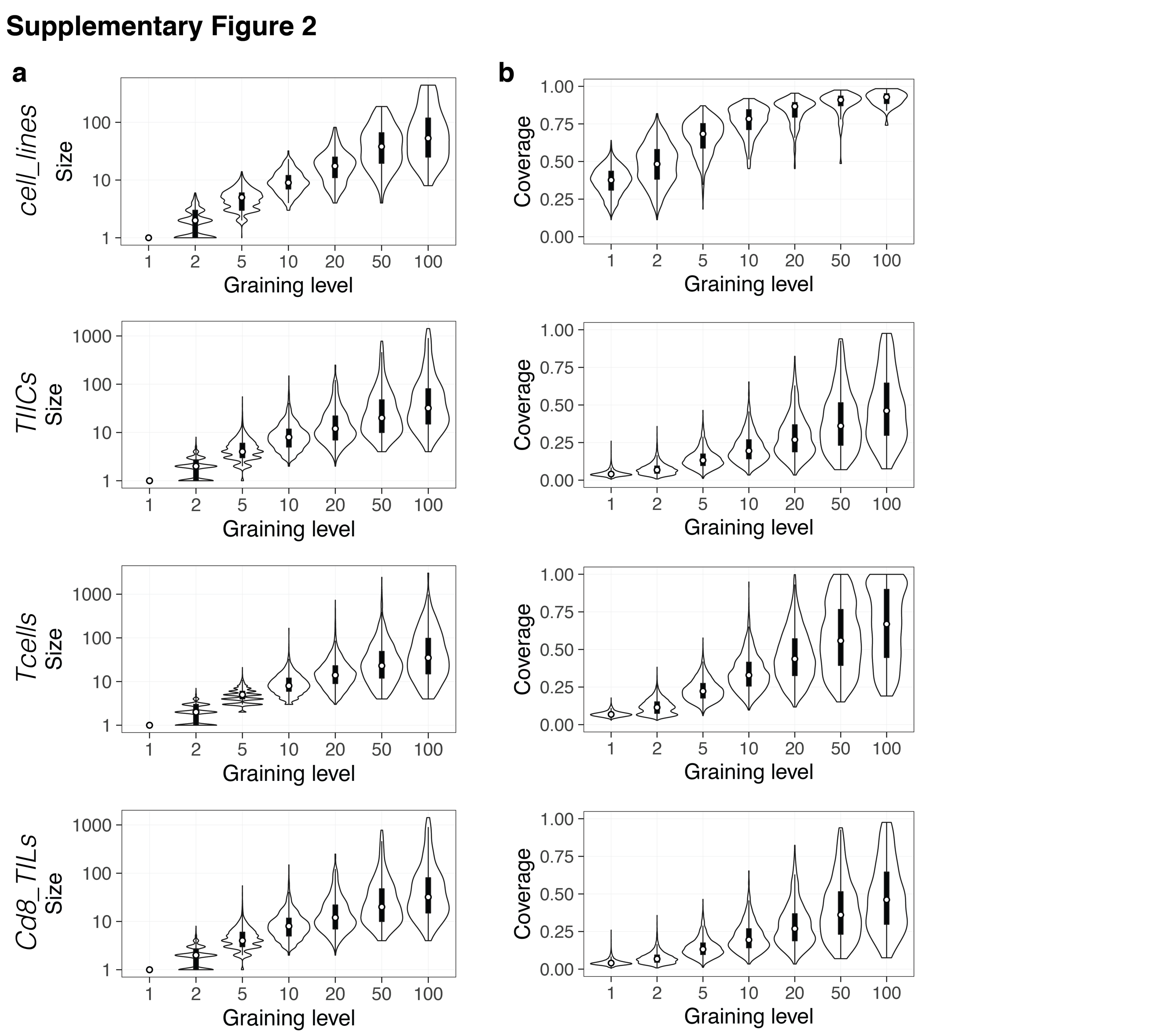


**Supplementary Figure 2. Metacell size and gene coverage distributions.**

**a**, Distribution of metacell sizes, defined as the number of single cells in each metacell. **b**, Distribution of metacell coverage, computed as the proportion of non-zero genes within each metacell. The white dots denote the median, and the boxes denote the interquartile range.


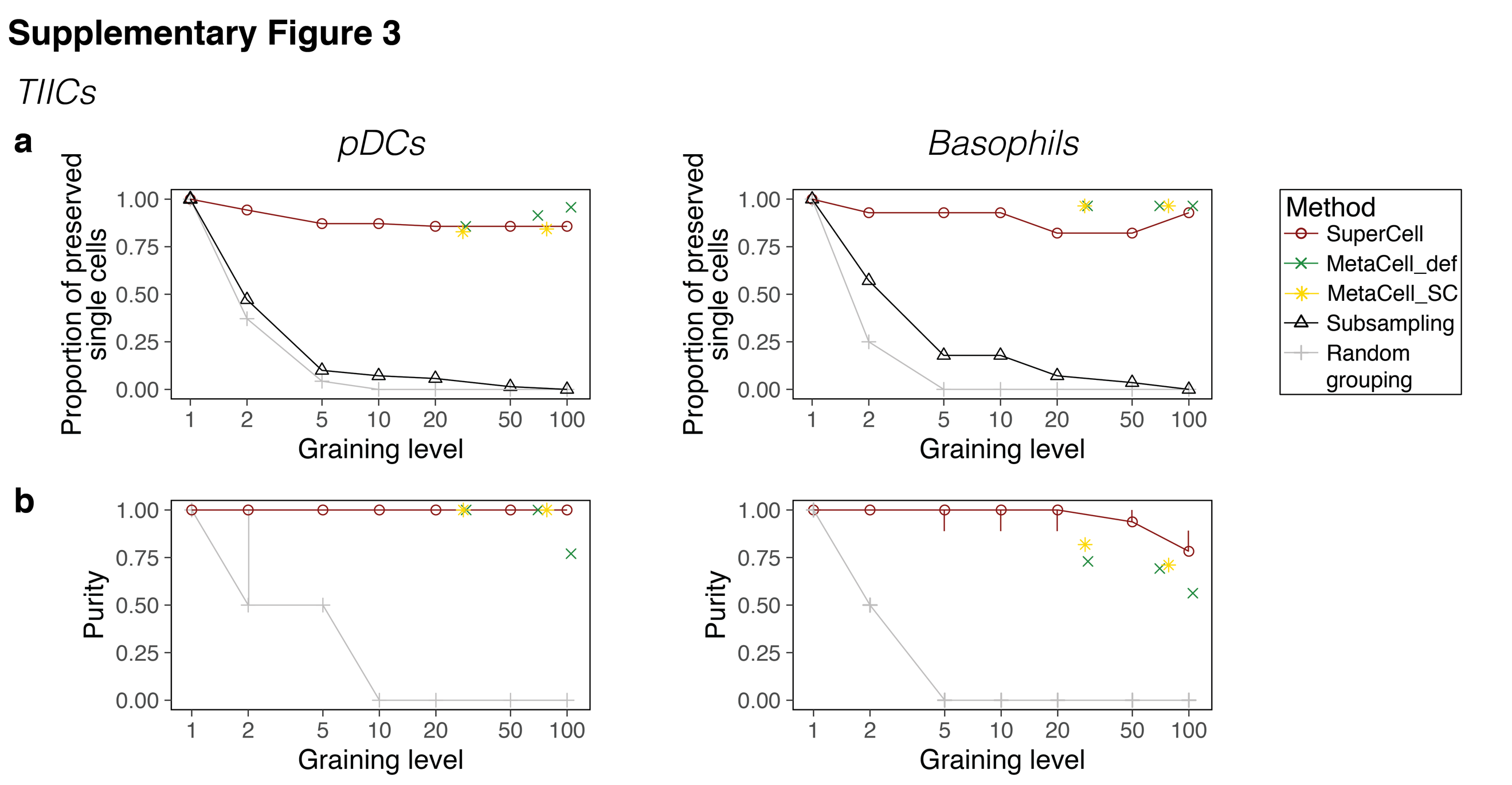


**Supplementary Figure 3. Metacells recover rare cell types.**

**a**, Proportion of correctly recovered single-cells from pDCs (left) and basophils (right), computed as a fraction of single cells of a particular cell types found in a metacell annotated to the same cell type. For the subsampling, it is computed as the proportion of subsampled single cells of the cell type of interest. **b**, Median purity of single cells within pDC metacells (left) or basophil metacells (right) (see Methods). Error bars denote the 1^st^ and 3^rd^ quartiles.


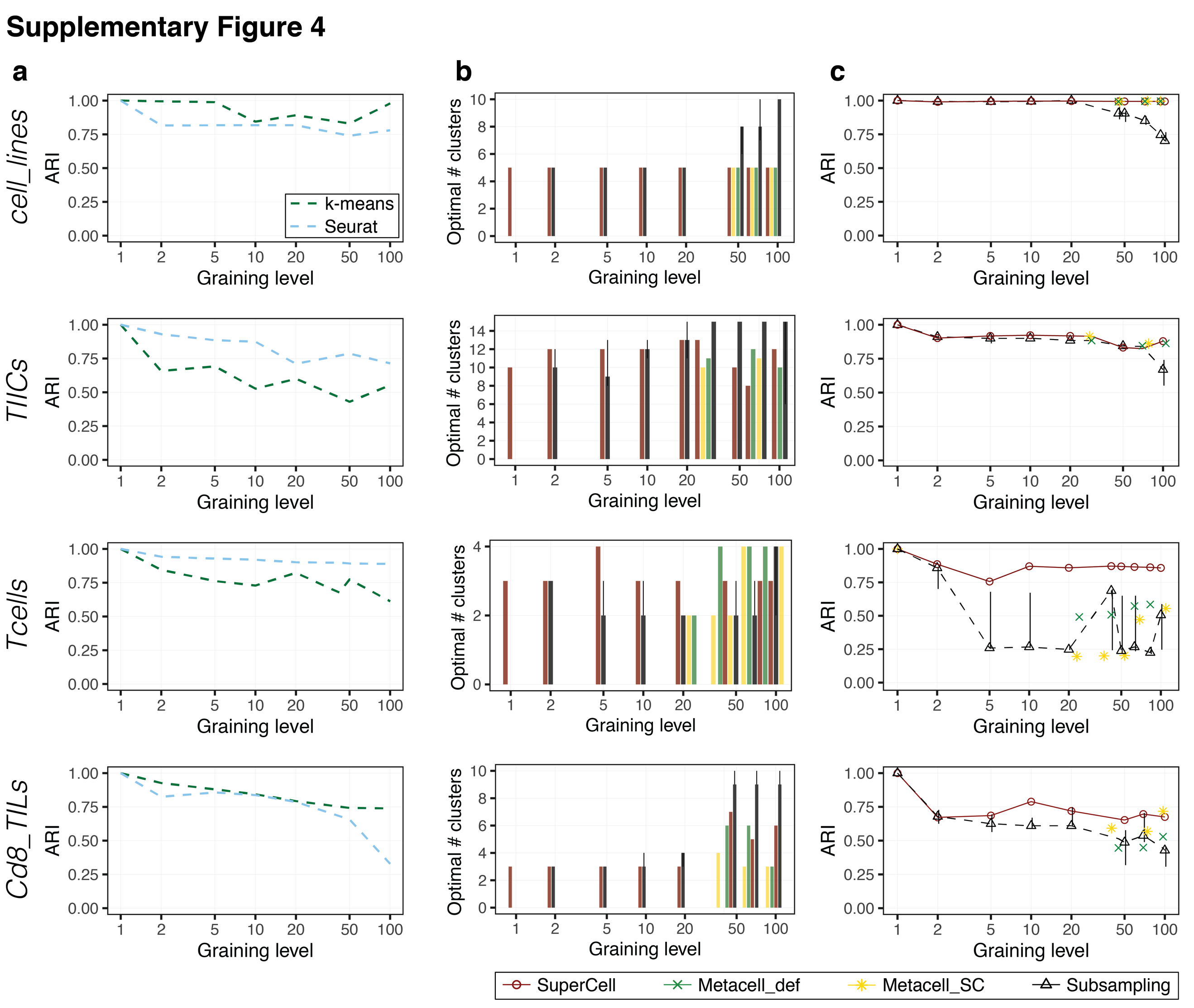


**Supplementary Figure 4. Metacells preserve clustering.**

**a**, Consistency of the metacell clustering obtained using k-means (green) and Seurat (blue) clustering algorithms. **b**, Optimal number of clusters based on the maximum silhouette coefficient. **c**, ARI values representing the consistency between clusters identified in metacells using the predicted optimal number of clusters (based on the maximum silhouette coefficient) and those identified in single cells.


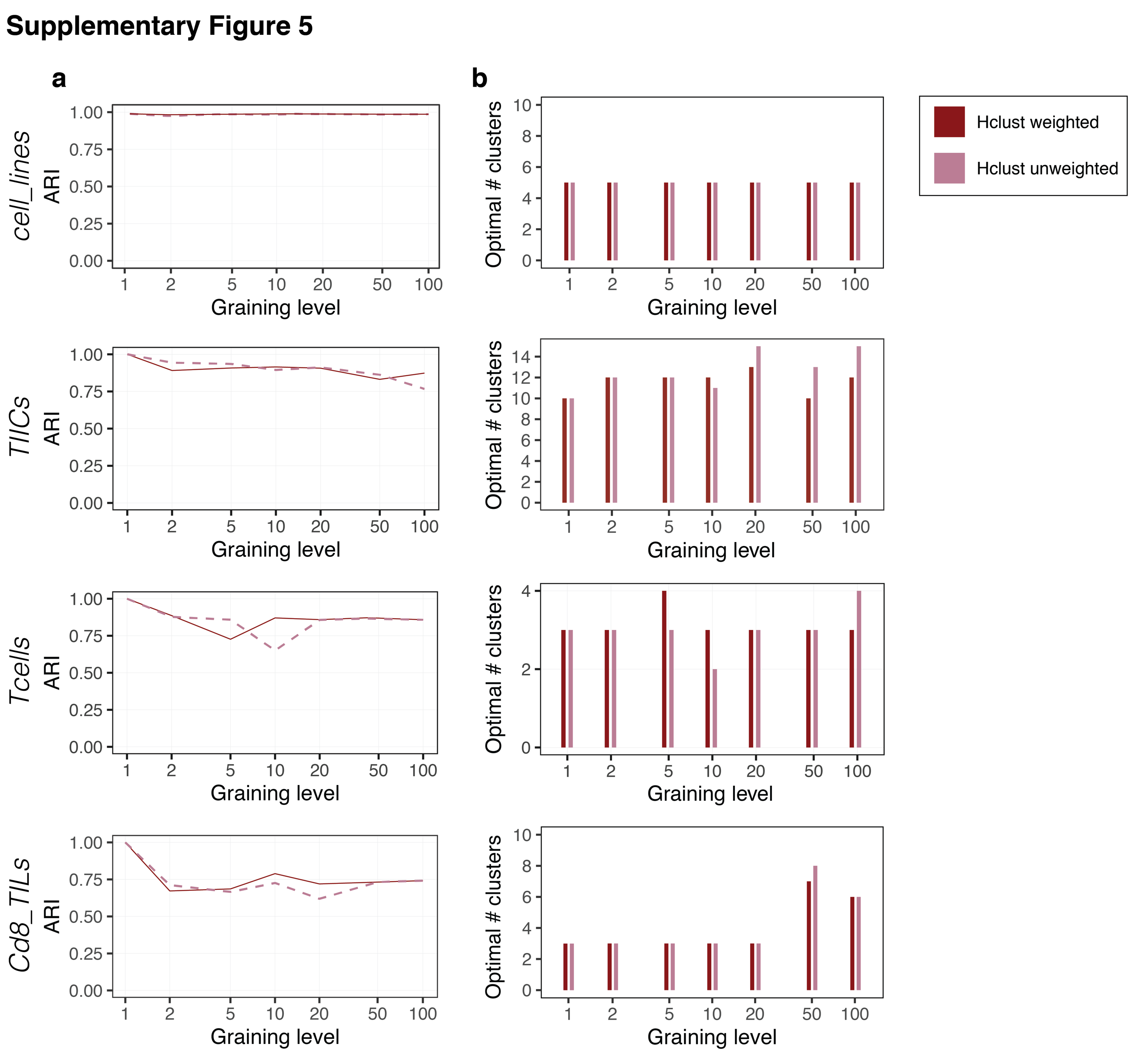


**Supplementary Figure 5. Metacells are compatible with unweighted clustering.**

**a**, Consistency of the metacell clustering obtained using weighted (solid dark red line) and unweighted (dashed orchid line) hierarchical clustering algorithms. **b**, Optimal number of clusters based on the maximum silhouette coefficient for the weighted (dark red) and unweighted (orchid) hierarchical clustering.


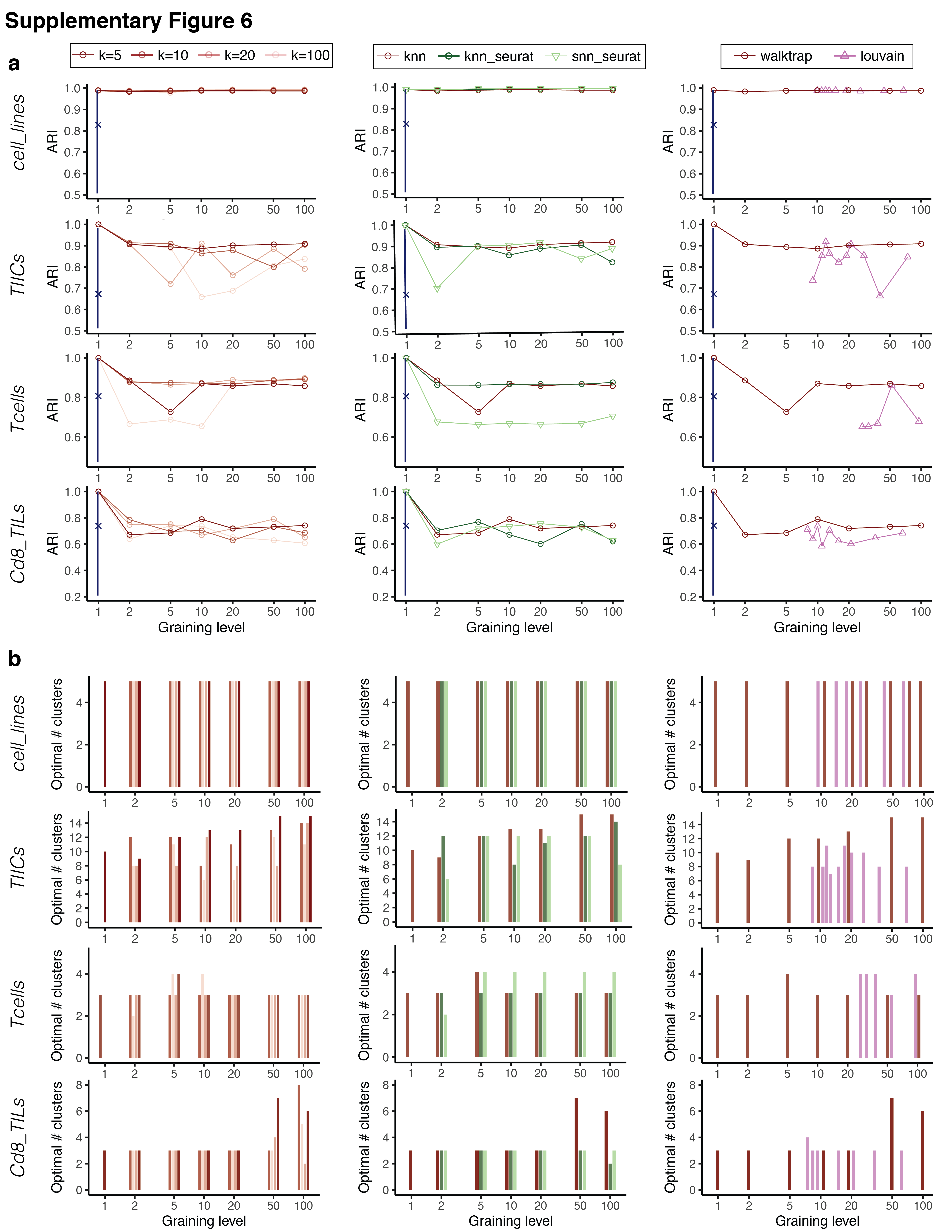


**Supplementary Figure 6. Clustering of metacells is robust to different ways of building metacells.**

Consistency of clustering (**a**) and optimal number of clusters (**b**) of metacells computed with different values for the parameter $k$ in kNN single-cell network (left), or different ways of the construction of single-cell network including Seurat kNN and Seurat sNN (shared nearest neighbors) (middle), or different ways of single-cell network clustering into metacells including Louvain algorithm for four datasets (right). The default parameters of the SuperCell algorithm are shown in dark red (i.e., kNN with $k=5$ and walktrap clustering). The blue line shows the range of ARI values when other clustering algorithms are applied to the single-cell data (median shown with “X”).


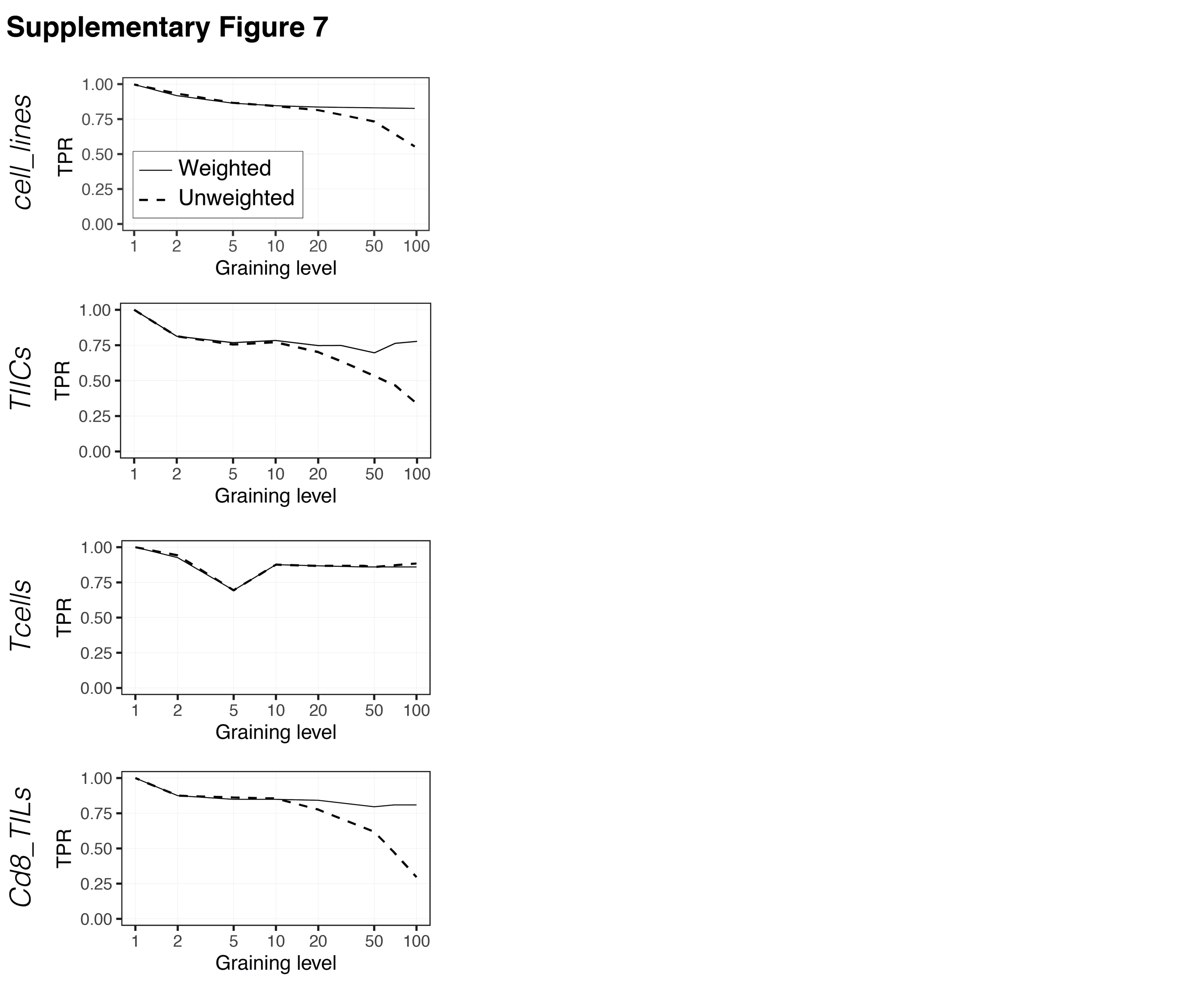


**Supplementary Figure 7. Metacells are compatible with unweighted differential expression analysis.**

Recovery of the cluster-specific differentially expressed genes using unweighted differential expression algorithm (i.e., unweighted t-test) for four datasets.


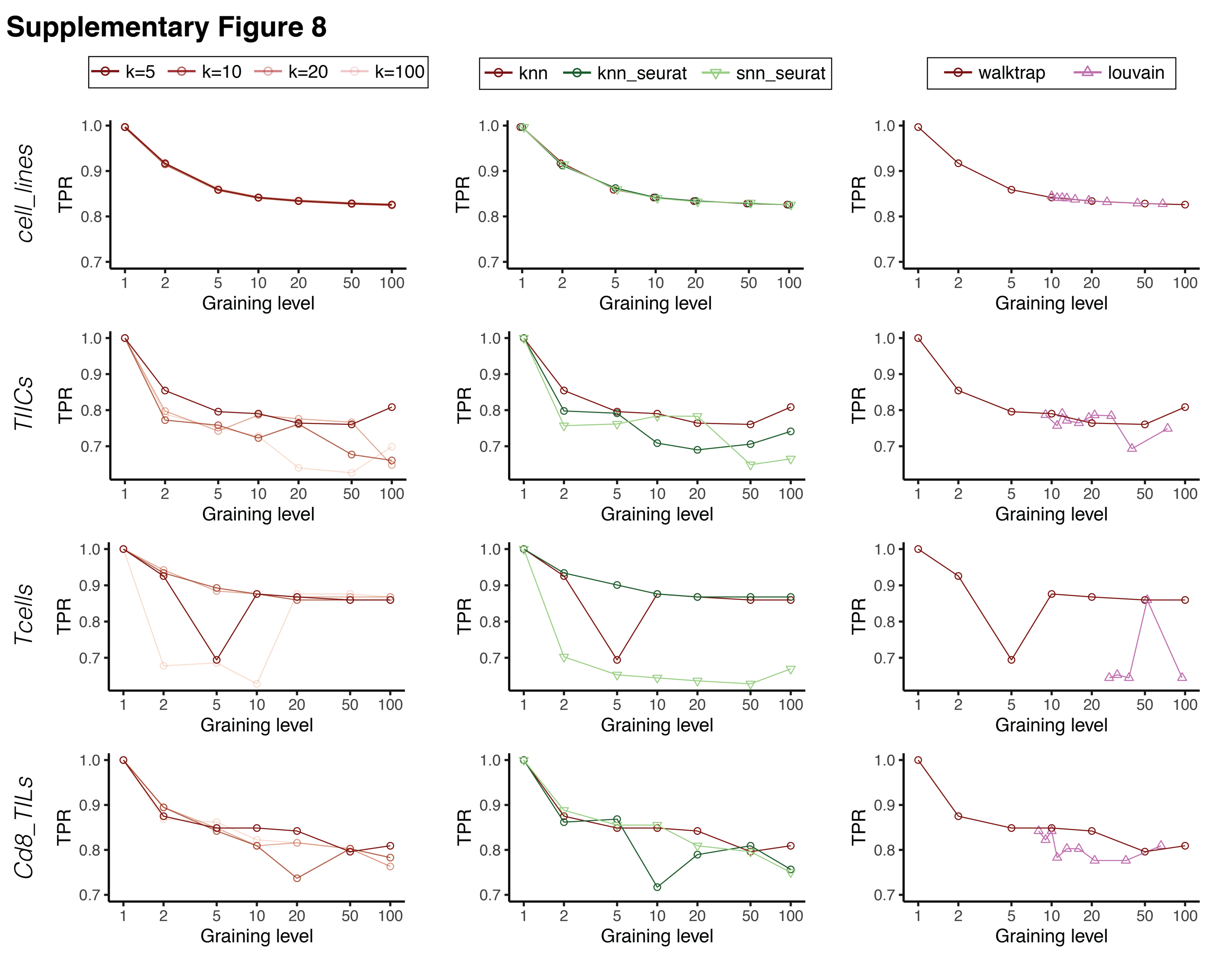


**Supplementary Figure 8. Differential expression of metacells is robust to different ways of building metacells.**

Recovery of cluster-specific differentially expressed genes of metacells computed with different values for the parameter $k$ in kNN single-cell network (left), or different ways of the construction of single-cell network, including Seurat kNN and Seurat sNN (shared nearest neighbors) (middle), or different ways of single-cell network clustering into metacells including Louvain algorithm for four datasets (right). The default parameters of the SuperCell algorithm are shown in dark red (i.e., kNN with $k=5$ and walktrap clustering).


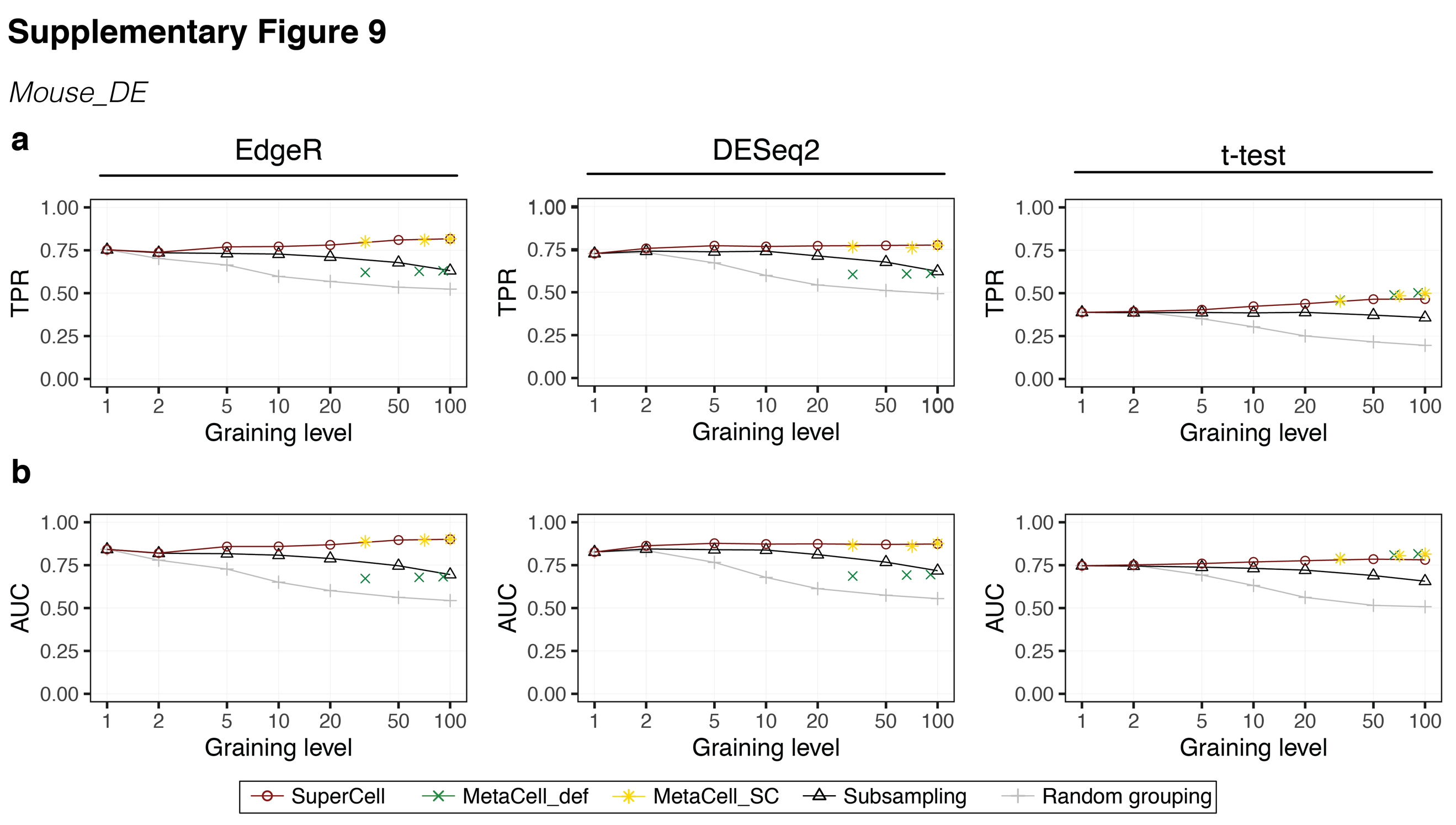


**Supplementary Figure 9. Metacells preserve differential expression between conditions.**

TPR (**a**) and AUC (**b**) of the recovery of differentially expressed genes between treated and control samples using EdgeR, DESeq2 and t-test approaches. The ground truth is the differential expression analysis from bulk RNA-seq.


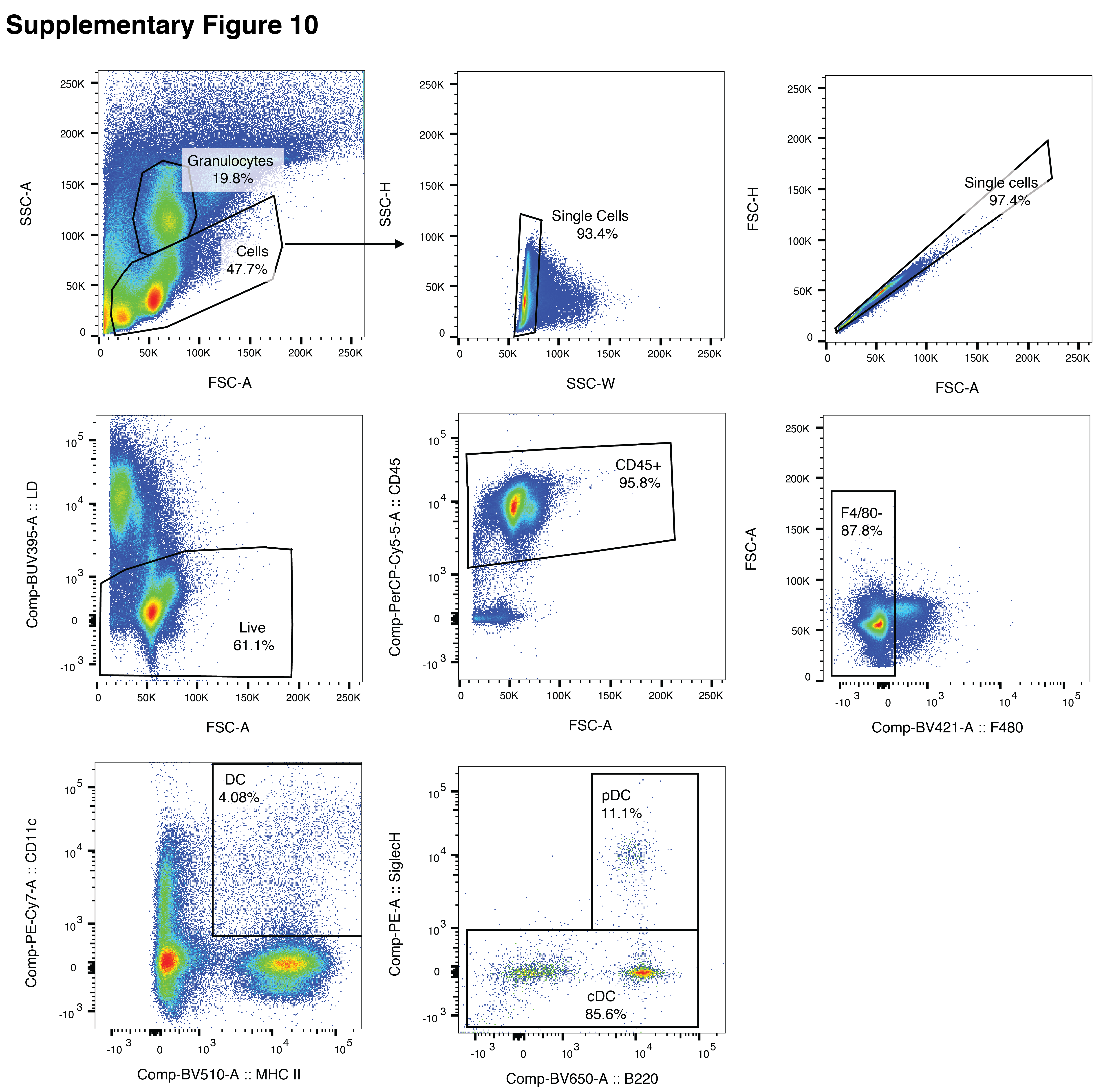


**Supplementary Figure 10. Gating strategy for the flow cytometry analysis of DCs from murine KP1.9 lung adenocarcinoma for one representative sample.**


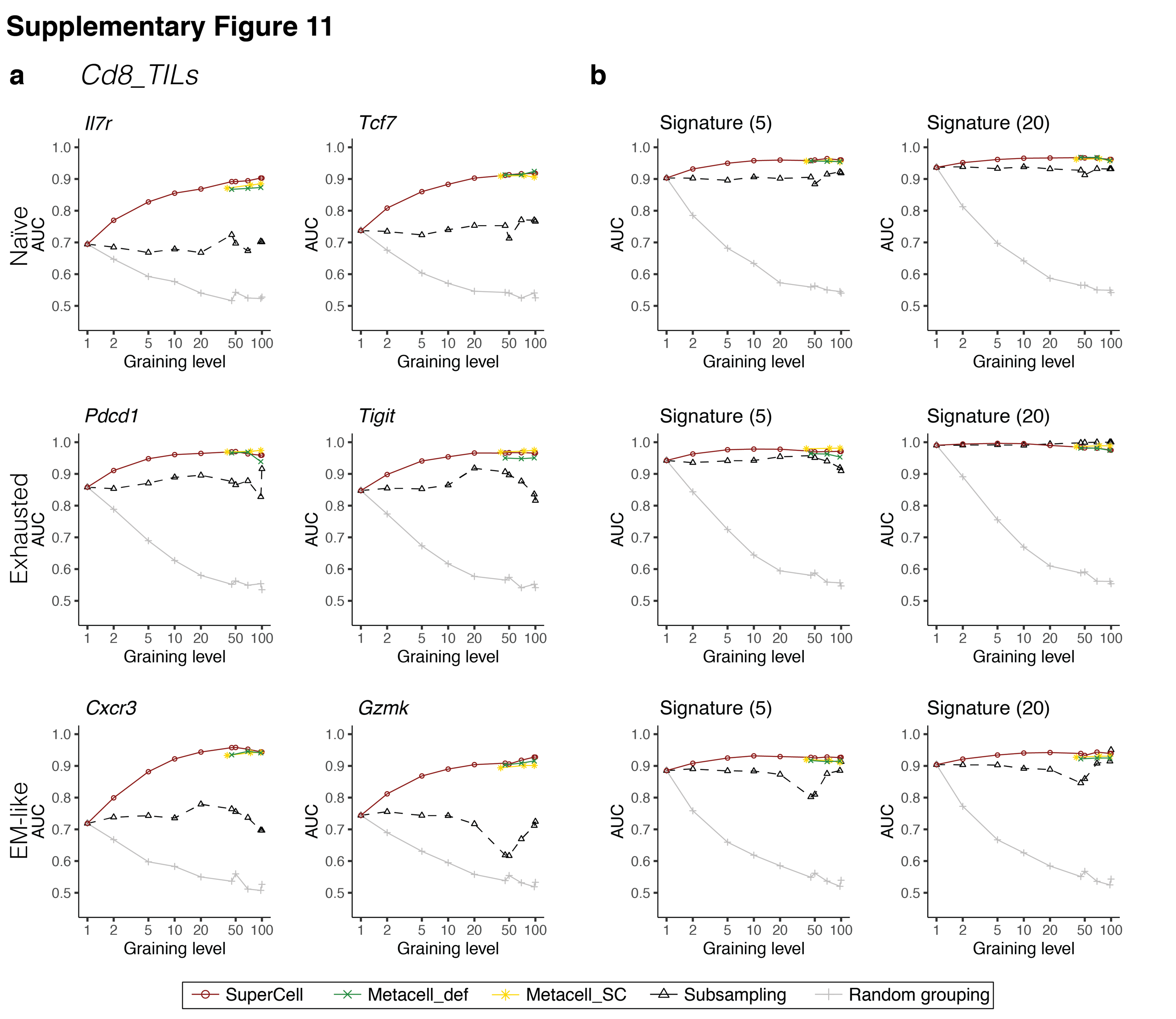


**Supplementary Figure 11. Cell type annotation in the *Cd8_TILs* dataset.**

**a**-**b**, AUC of the recovery of naïve (top), exhausted/progenitor exhausted (middle) and effector memory-like (bottom) CD8 T cells from the *TILs* dataset using single markers (**a**) or signatures defined from the same dataset (**b**) that consists of the top 5 or 20 upregulated genes.


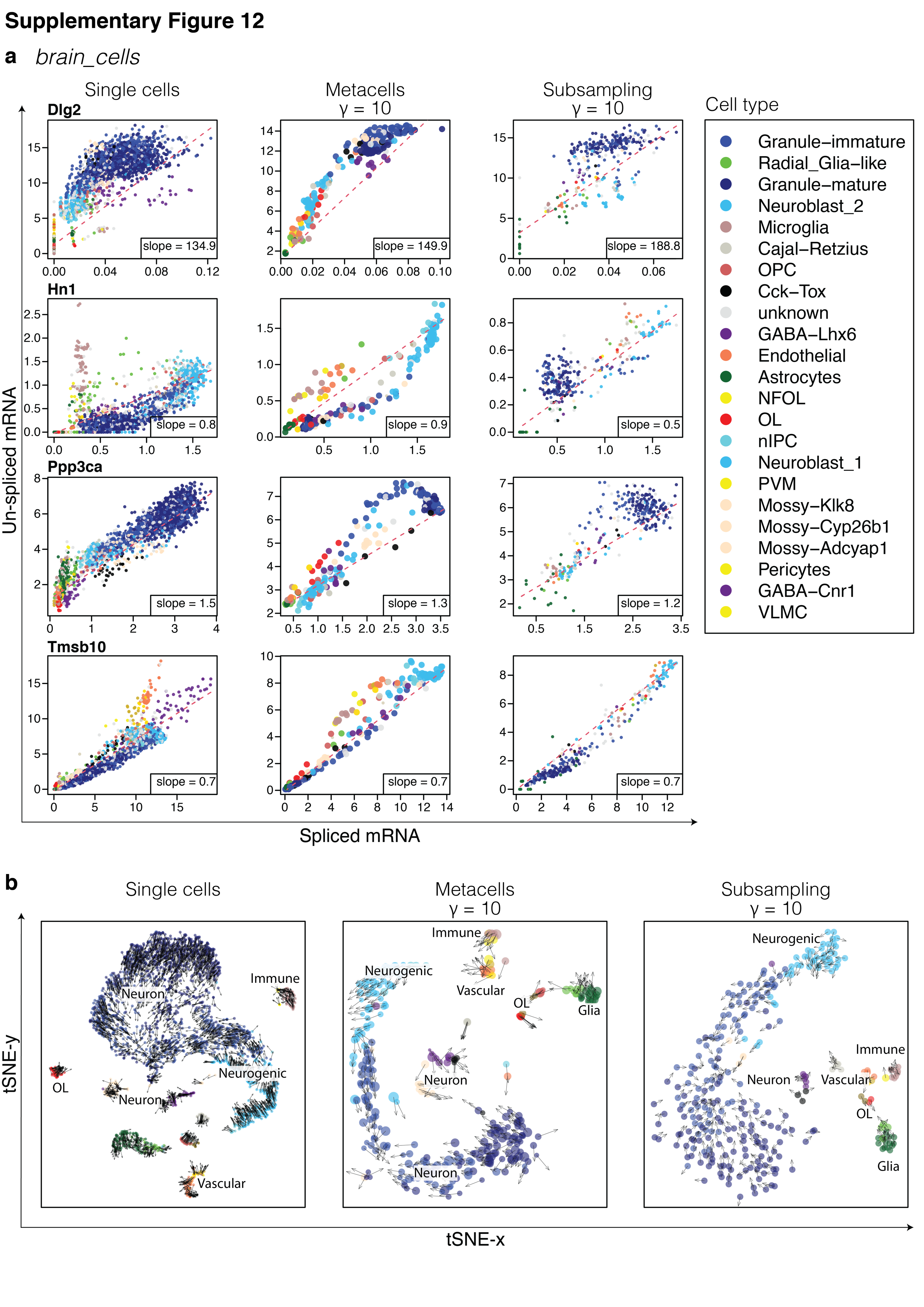


**Supplementary Figure 12. Conservation of RNA velocity results in the *brain_cells* dataset.**

**a**, Spliced/un-spliced phase portraits and estimated equilibrium slopes (red dashed lines) for the single cells (left), metacells ($\gamma=10$) (middle) and subsampling ($\gamma=10$) (right) in the *brain_cell* dataset. **b**, Separate tSNE visualization of RNA velocity for the single cells (left), metacells ($\gamma=10$) (middle) and subsampling ($\gamma=10$) (right). Colors indicate the cell type annotation of single-cell data and metacells are colored according to the majority of cells in each metacell.


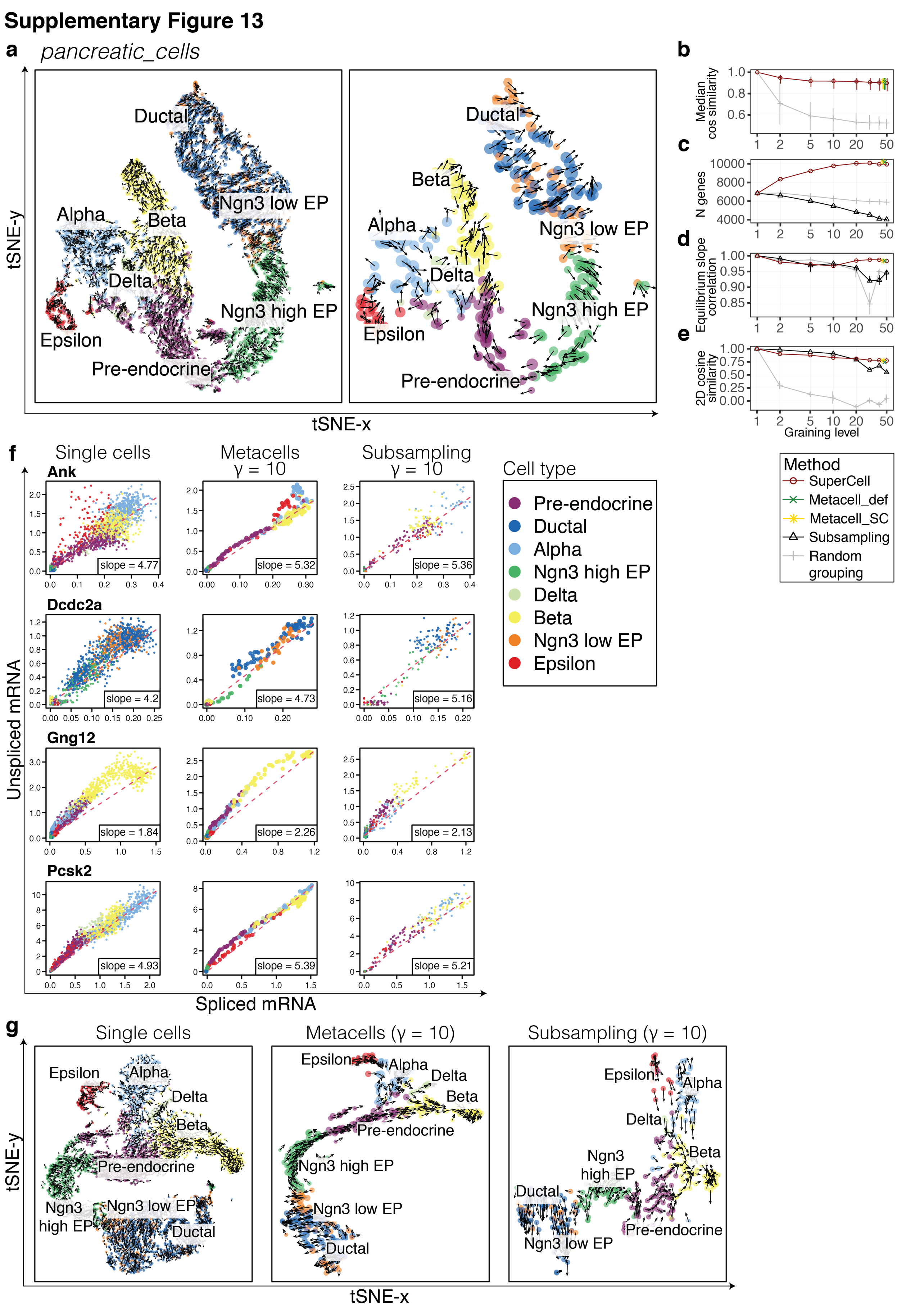


**Supplementary Figure 13. Conservation of RNA velocity results in the *pancreatic_cells* dataset.**

**a**, Joint tSNE visualization of RNA velocity for single cells (left) and metacells ($\gamma=10$) (right) in the *pancreatic_cells* dataset ($N=3’696$). Colors indicate the cell type annotation of single-cell data and metacells are colored according to the majority of cells in each metacell. **b**, Median purity of metacell velocity computed as a cosine similarity of velocities within each metacell. **c**, Number of genes with valid estimated equilibrium slope values. **d**, Median Pearson correlation of gene equilibrium slope values obtained in single cells and metacells. **e**, Median similarity of 2D RNA velocities computed as a cosine similarity between a 2D RNA velocity of each single cell and a 2D RNA velocity of the metacell it belongs to. **f**, Spliced/un-spliced phase portraits and estimated equilibrium slopes (red dashed lines) for the single cells (left), metacells ($\gamma=10$) (middle) and subsampling ($\gamma=10$) (right). **g**, Separate tSNE visualization of RNA velocity for the single cells (left), metacells ($\gamma=10$) (middle) and subsampling ($\gamma=10$) (right).


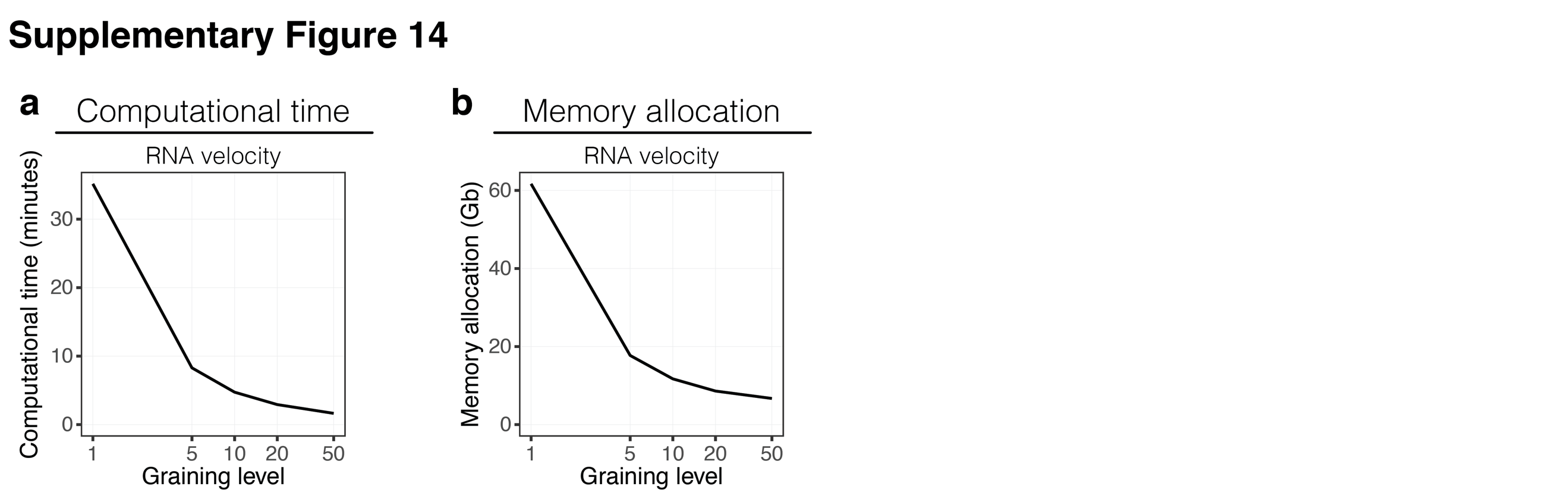


**Supplementary Figure 14. Computational time and memory allocation for RNA velocity.**

**a**-**b**, Computational time (**a**) and memory allocation (**b**) for the RNA velocity over different graining levels for the *brain_cells* dataset ($N=3’396$).


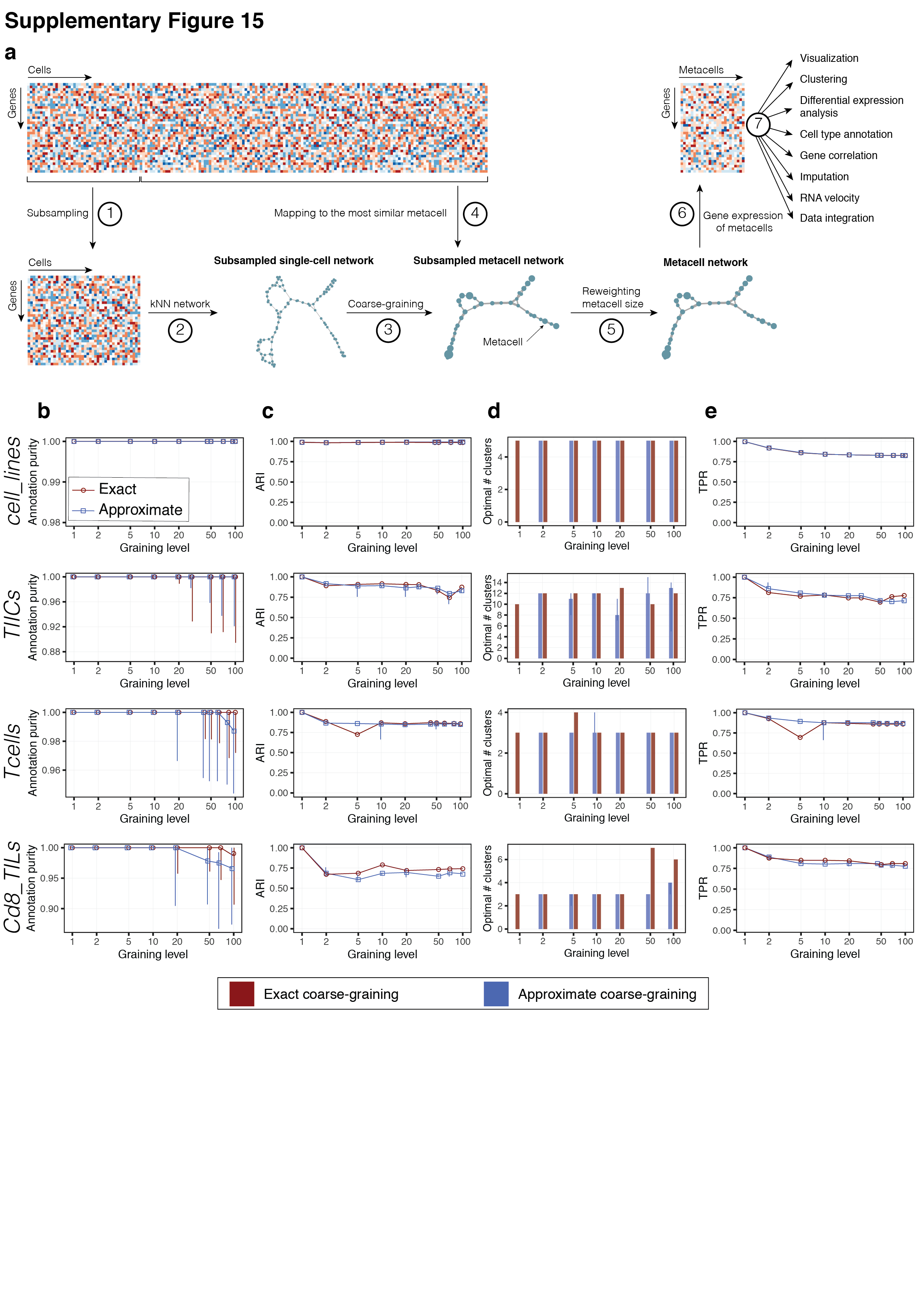


**Supplementary Figure 15. Approximate coarse-graining in SuperCell.**

**a**, Overview of the approximate coarse-graining approach. (1) A subset of cells is randomly selected. (2) The single-cell network is constructed from the subsampled single-cell gene expression matrix using k-nearest neighbors (kNN) algorithm. (3) The metacell network is constructed by grouping similar cells into metacells at a user-defined graining level ($\gamma$). (4) Remaining cells are mapped to the most similar metacell. (5) The metacell network is reweighted. (6) The gene expression matrix of metacells is computed by averaging gene expression within each metacell. (7) This metacell gene expression matrix can be used for visualization and downstream analyses such as clustering, differential expression, cell type annotation, gene correlation, RNA velocity and data integration. **b-d**, Comparison of the results of the exact and the approximate coarse-graining in terms of metacell purity (**b**), consistency of clustering (**c**), optimal number of clusters (**d**) and recovery of cluster-specific differentially expressed genes (**e**) for the exact (red) and the approximate (blue) coarse-graining. For the approximate coarse-graining, the center of the error bars denotes the median, and the extrema denote the 1^st^ and 3^rd^ quartiles (obtained with different random seeds).


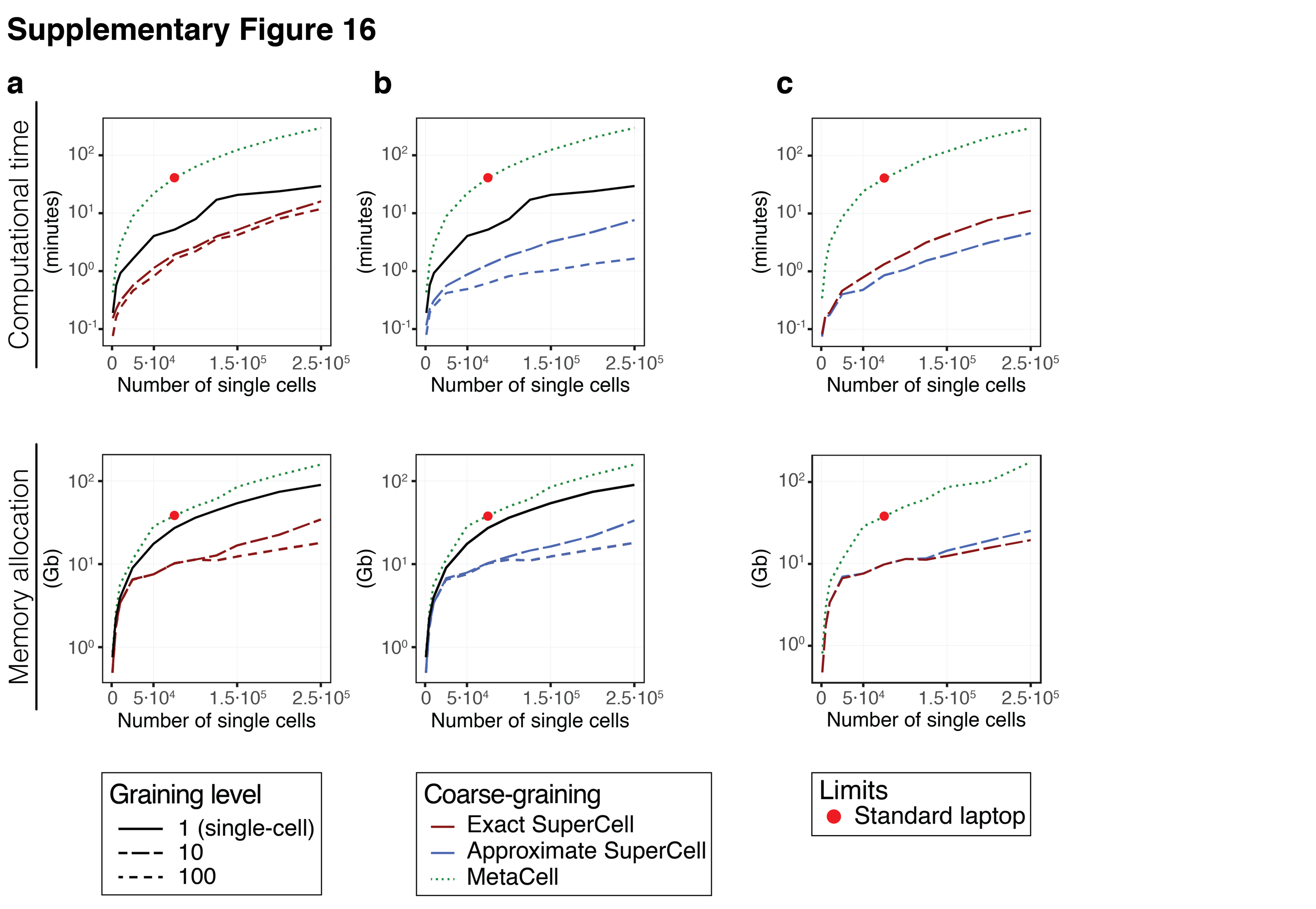


**Supplementary Figure 16. Computational time and memory allocation for metacell construction and downstream analyses.**

**a**,**b**, Computational time (top) and memory allocation (bottom) for the building of metacells with MetaCell, exact (**a**) or the approximate (**b**) SuperCell followed by downstream analyses including dimensionality reduction, clustering and DE analysis for the metacell and the single-cell data on a single dataset (extracted from GSE136831, see Methods). **c**, Computational time (top) and memory allocation (bottom) for the building of metacells with MetaCell or the exact and approximate SuperCell. Red dots represent the limits reached on standard desktops (16G of RAM).

**Supplementary Table 1. Datasets used for the analysis.**

**Supplementary Table 2. Datasets integrated in the *TIM_atlas* dataset.**

**Supplementary Table 3. Genes ranked better in differential expression analysis (cDC vs pDC) at metacell level.**

**Supplementary Table 4. Genes ranked better in differential expression analysis (pDC vs cDC) at metacell level.**

**Supplementary Table 5. Antibodies used in flow cytometry (Fig. 2f, Supplementary Fig. 10).**
